# Expression of HLA-DR in Cytotoxic T Lymphocytes: A Validated Predictive Biomarker and a Potential Therapeutic Strategy in Breast Cancer

**DOI:** 10.3390/cancers13153841

**Published:** 2021-07-30

**Authors:** Diana P. Saraiva, Sofia Azeredo-Lopes, Ana Antunes, Rute Salvador, Paula Borralho, Beatriz Assis, Isabel L. Pereira, Zita Seabra, Ida Negreiros, António Jacinto, Sofia Braga, M. Guadalupe Cabral

**Affiliations:** 1 iNOVA4Health, CEDOC, NOVA Medical School (NMS), Universidade Nova de Lisboa, 1150-082 Lisbon, Portugal; diana.saraiva@nms.unl.pt (D.P.S.); rute.salvador@nms.unl.pt (R.S.); antonio.jacinto@nms.unl.pt (A.J.); sofia.braga@cuf.pt (S.B.); 2 Public Health and Biostatistics Department, NOVA Medical School (NMS), Universidade Nova de Lisboa, 1150-082 Lisbon, Portugal; sofia.azeredo@nms.unl.pt; 3 CHRC, CEDOC, NOVA Medical School (NMS), Universidade Nova de Lisboa, 1150-082 Lisbon, Portugal; ana.antunes@nms.unl.pt; 4 Unidade de Mama, Instituto CUF de Oncologia, 1998-018 Lisbon, Portugal; paula.b.nunes@jmellosaude.pt (P.B.); beatriz.assis@cuf.pt (B.A.); isabel.pereira@jmellosaude.pt (I.L.P.); ida.negreiros@jmellosaude.pt (I.N.); 5 Instituto de Anatomia Patológica, Faculdade de Medicina da Universidade de Lisboa, 1649-028 Lisbon, Portugal; 6 Unidade de Imagiologia, Hospital Vila Franca de Xira, 2600-009 Vila Franca de Xira, Portugal; zita.seabra@hvfx.pt

**Keywords:** breast cancer, cytotoxic T lymphocytes, HLA-DR, biomarker, neoadjuvant chemotherapy, 3D co-cultures, immune-modulation

## Abstract

**Simple Summary:**

More than 50% of breast cancer (BC) patients selected for neoadjuvant chemotherapy (NACT) are subjected to at least a 6-month regimen of this treatment without a clear benefit, probably delaying more effective therapeutic strategies and being exposed to potential treatment-associated toxicity. Thus, it is urgent to implement reliable predictive biomarkers, as well as novel treatments for NACT non-responder patients. This study validates that the HLA-DR level in cytotoxic T lymphocytes (CTLs) is an independent and robust predictive factor of BC patients’ response to NACT, as previously proposed. Hence, a predictive probability model of response was developed as a new tool to improve treatment decisions. HLA-DR level in CTLs also have a general prognostic value, which might be relevant for long-term BC management. In addition, our results suggest that increasing the expression of HLA-DR in CTLs of non-responders could be a promising therapeutic strategy to ameliorate BC response to NACT.

**Abstract:**

Neoadjuvant chemotherapy (NACT) is common in breast cancer (BC) treatment, though more than half of the patients lack an effective response. Therefore, new predictive biomarkers and alternative therapies are crucial. Previously, we proposed HLA-DR-expressing cytotoxic T lymphocytes (CTLs) as a potential biomarker of the response to NACT. To validate this observation and further investigate these cells, 202 BC patients were enrolled. Flow cytometry analyses were performed in 61 biopsies and 41 blood samples pre-NACT and 100 non-NACT tumor samples. All the patients were followed up for 34 months. Blood-isolated immune cells were cultured with BC cell lines in a 3D system. We confirmed that HLA-DR level in CTLs is a highly sensitive, specific, and independent biomarker to predict response to NACT and developed a predictive probability model. This biomarker was also associated with progression-free survival, regardless of the treatment. The clinical observations are substantiated by the anti-tumor properties of HLA-DR-expressing CTLs. Intriguingly, HLA-DR level in CTLs can be modulated ex vivo, boosting their capacity to kill tumor cells synergistically with doxorubicin. Thus, HLA-DR expression in CTLs is a validated tool to select patients that will actually benefit from NACT, and its stimulation might be a novel therapeutic approach for BC.

## 1. Introduction

Breast cancer (BC) is the most frequent type of cancer in women worldwide, accounting for up to two million new cases per year [1]. Early-stage disease has a high 5-year survival rate, with 99% for estrogen receptor positive (ER+) tumors, 94% for human epidermal growth factor receptor 2 (HER2+) overexpressing tumors, and 85% for triple negative breast cancer (TNBC) [2]. Nevertheless, when the disease is in an advanced stage, the survival rate is lower, probably due to the lack of effective specific treatment options [2]. Indeed, independently of the BC subtype, the treatment option for locally advanced BC is neoadjuvant chemotherapy (NACT). Although NACT is important in downstaging the tumor, allowing a breast-conserving surgery [3], approximately half of the patients do not respond to this treatment [4,5]. Thus, it is essential to find biomarkers of response to NACT, as well as alternative therapeutic options for non-responders.

NACT efficacy may depend on immune players present in the tumor microenvironment, namely, tumor-infiltrating lymphocytes (TILs) and, as such, TILs have been studied as possible biomarkers to predict response to NACT. Actually, chemotherapy promotes immunogenic cell death (ICD), which involves the release of damage-associated molecular patterns (DAMPs) by dying cells that might lead to the activation of particular TILs (namely cytotoxic T lymphocytes—CTLs) that, in turn, will allow the control of NACT-resistant tumor cells [6]. However, accumulating evidence has been shown that the same ICD-resulting DAMPs could, in certain conditions, support cancer progression and resistance to treatment [7]. Additionally, tumors have several mechanisms to escape immune surveillance and TILs-mediated killing [8]. Hence, quantification of TILs is still not used routinely in the clinical setting.

We have previously reported that CTLs expressing high levels of the activation marker HLA-DR in fresh BC biopsies were a highly sensitive and specific factor to predict response to NACT [9]. In this preceding study, we found that HLA-DR+ CTLs were mainly present inside the tumor microenvironment; produced high levels of cytotoxicity-related molecules, such as IFN-γ, Granzyme B, and Perforin; were negatively correlated with immunosuppressive features of the tumor milieu; and were reflected systemically [9].

HLA-DR (human leukocyte antigen) is a class II MHC molecule, normally expressed in professional antigen presenting cells. Nonetheless, in lymphocytes, namely in CTLs, it is a late-phase activation marker, being upregulated 24/48 h after the activation of these cells, and it is associated with increased IFN-γ production. It is preceded by an increase in the expression of CD69 and CD25, which are upregulated 4 h and 12 h after T cell activation, respectively [10,11,12,13,14].

HLA-DR expression on effector T lymphocytes upon their activation has been intensively described in some diseases, such as auto-immune diseases and viral infections [15,16,17]. The increase of HLA-DR at CTLs’ surface, upon stimulation, could also be required to boost an effective immune response. Indeed, HLA-DR+ CTLs were found to have the machinery needed for antigen processing and loading on HLA-DR molecules and, additionally, could express CD86 and CD80, which are the co-stimulatory molecules of antigen presenting cells that are necessary for the proper T cell effector function [18]. Moreover, it was described that T cell–T cell synapsis occurs to allow T cells to secrete IFN-γ towards each other, compelling the differentiation of more protective T cells [19]. These T cell–T cell interactions and mutual antigen presentation can be essential for mounting a suitable anti-tumor response.

In order to confirm HLA-DR-expressing CTLs as a biomarker of response to NACT, following the REMARK (reporting recommendations for tumor marker prognostic studies) criteria [20], we conducted a validation study in an independent cohort of BC patients, tackling one of the clinical needs in BC management. Moreover, we took advantage of a 3D co-culture platform of BC cell lines and patient-derived immune cells, developed to be used in the future as a drug screening platform [21], to shed some light on the anti-tumor role of HLA-DR+ CTLs and further explore their potential therapeutic value.

Thus, here we validate HLA-DR level in CTLs as a new, independent biomarker to predict BC response to NACT and, additionally, as a general BC prognosis factor. Likewise, we suggest that the modulation of HLA-DR expression in CTLs could be an interesting approach to ameliorate advanced BC treatment and, therefore, the survival rates of this disease.

## 2. Materials and Methods

### 2.1. Patients’ Samples

**Figure 1:**
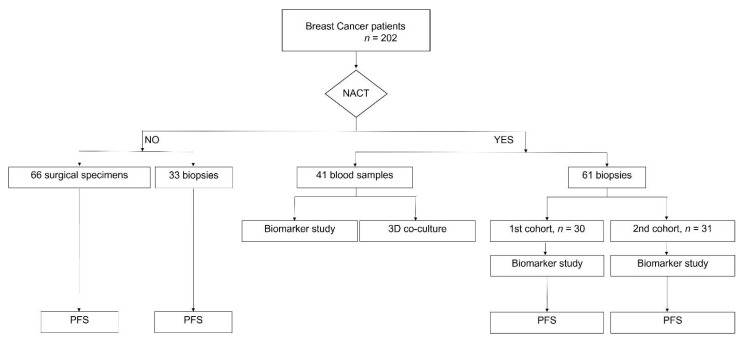
Flowchart of patients enrolled in this study. Breast cancer patients (*n* = 202) were divided according to the prescription of neoadjuvant chemotherapy (NACT). The biopsies of NACT patients were divided into two different cohorts for the predictive biomarker study. Blood samples were also used in this study and to isolate PBMCs for in vitro 3D co-culture assay. Progression-free survival (PFS) was performed for NACT and non-NACT patients.

This prospective study was designed following the REMARK criteria [20]. A total of 202 breast cancer (BC) patients were enrolled in this study (Figure 1). Sixty-one fresh biopsies (from a previous pilot cohort and from this validation cohort) of BC patients selected for neoadjuvant chemotherapy (NACT) were collected in Transfix (Cytomark, Buckingham, UK) to preserve cellular antigens. NACT was composed of four cycles of doxorubicin and cyclophosphamide every 3 weeks, followed by 12 weeks of paclitaxel. Trastuzumab was given every 3 weeks to HER2+ patients. A summary of the patients’ characteristics is presented in Table 1. The biopsies were mechanically dissociated with a BD Medicon (BD Biosciences, Franklin Lakes, NJ, USA), filtered through a 30 μm mesh (Sysmex, Kōbe, Japan), washed with PBS1X, and stained for flow cytometry (as described below). Biopsies and surgical specimens from non-NACT patients were similarly handled and were used, together with NACT samples, in the progression-free survival analysis. Forty-one blood samples were collected from non-matched BC patients selected for NACT in Vacutainer tubes with EDTA (BD Biosciences, Franklin Lakes, NJ, USA). Whole blood staining for flow cytometry was performed as described below. Peripheral blood mononuclear cells (PBMCs) were isolated from whole blood by a Ficol gradient (Merck Millipore, Burlington, VT, USA) and cryopreserved in 90% fetal bovine serum (FBS, Biowest, Riverside, CA, USA) and 10% dimethyl sulfoxide (DMSO, Sigma Aldrich, St. Louis, MO, USA) to be used later in the 3D co-culture systems. Isolated PBMCs from eight healthy donors’ buffy coats provided by Instituto Português do Sangue e da Transplantação were used specifically in the cytotoxicity assay.

Samples were gathered from Hospital CUF Descobertas, Hospital de Vila Franca de Xira, Hospital Prof. Doutor Fernando Fonseca, and Hospital Santa Maria. For each patient, written informed consent and approval by the ethical committees of the hospitals and of the NOVA Medical School (97/2019/CEFCM) were obtained.

### 2.2. Flow Cytometry

Cell suspension from the processed BC samples was stained with a cocktail of mouse anti-human surface antibodies for 15 min. The cells were then fixed and permeabilized with Fix/Perm kit (Invitrogen, Waltham, MA, USA), followed by intracellular staining for 30 min. In case of whole blood, the staining protocol was similar, but it was followed by a step of red blood cells lysis with RBC lysis buffer (Biolegend, San Diego, CA, USA). Data were acquired in BD FACS Canto II (BD Biosciences, Franklin Lakes, NJ, USA), and the results were analyzed using FlowJo software v10. The data are presented as a percentage of the populations with respect to the single cells’ gate (Figure S1). To analyze the expression levels of HLA-DR in cytotoxic T lymphocytes (CTLs), we considered the median fluorescent intensity (MFI) (arbitrary units from the FACS) of the positive population and normalized it to the MFI of the negative population, as we previously described [9]. The negative population was superimposed with the unstained control. For the characterization of HLA-DR+ CTLs (Section 3.3.), we have quantified the percentage of each marker gated inside HLA-DR+ CTLs (Figure S1) and normalized it to the percentage of the same marker in HLA-DR negative CTLs to decrease variability between samples.

The monoclonal mouse anti-human antibodies used were: anti-CD45-PercP, anti-CD3-APC, anti-CD19-PE, anti-CD15-PE, anti-CD161-FITC, anti-CD4-FITC, anti-CD8-PE, anti-HLA-DR-APC, anti-CD127-PE-Cy7, anti-CD25-PE, anti-CD163-PE, anti-CD206-APC-Cy7, anti-PD-L1-APC, anti-CD11b-FITC, anti-IL-10-FITC, anti-CD69-PercP, anti-IFN-γ-APC-Cy7, anti-GranzymeB-FITC, anti-Ki67-PE, anti-CD45RO-PercP, anti-PD-1-FITC, anti-Tim3-APC-Cy7, anti-CD39-BV421 (Biolegend, San Diego, CA< USA), and anti-CD103-PE-Cy7 (Invitrogen, Waltham, MA, USA).

### 2.3. Fluorescence Activated Cell Sorting (FACS)

PBMCs from healthy donors were cultured overnight in RPMI-1640 (Gibco, Waltham, MA, USA) supplemented with 10% FBS and 1% Penicillin/Streptomycin (GE Healthcare, Chicago, IL, USA) and stimulated with 35 ng/mL of phorbol 12-myristate 13-acetate (PMA, Sigma Aldrich, St. Louis, CA, USA) and 1 µg/mL of ionomycin (Merck Millipore, Burlington, VT, USA) at 37 °C, 5% CO_2_. Cells were then stained with BD Horizon™ Fixable Viability Stain 450 (BD Biosciences, Franklin Lakes, NJ, USA) for 20 min in ice, followed by staining with the antibodies anti-CD45-PercP, anti-CD8-PE, and anti-HLA-DR-APC. Cells were sorted into two populations: CD45+/CD8+/HLA-DR+ and CD45+/CD8+/HLA-DRnegative in a FACS Aria III (BD Biosciences, Franklin Lakes, NJ, USA) with an efficiency above 90%.

### 2.4. Cytotoxicity Assays in 3D Co-Cultures

The BC cell lines MCF-7 and MDA-MB-231 were cultured in DMEM (Biowest, Riverside, CA, USA) supplemented with 10% FBS and 1% Penicillin/Streptomycin. Then, 3D co-culture in agarose-coated plates of both cell lines with patient-derived PBMCs in a 1:1 ratio was performed as previously described [21]. In some experiments, before the co-culture, PBMCs from NACT-responders and non-responders were stimulated with PMA/ionomycin as above described or by T cell receptor (TCR) engagement with mouse anti-human anti-CD3 (5 μg/mL), anti-CD28 (1 μg/mL), and 5 μg/mL of the crosslinking antibody rat anti-mouse IgG1 (Biolegend, San Diego, CA, USA). Forty-eight hours after the start of the co-culture, doxorubicin (Sigma Aldrich, St. Louis, CA, USA) was also added at 0.1 μg/mL or 0.5 μg/mL, and DMSO was used as a negative control. Four days after the start of the co-culture (or 48 h after the addition of doxorubicin when used), all spheroids were removed from the plate, dissociated by pipetting, and stained with the viability dye to evaluate, by flow cytometry, the percentage of viable cancer cells in the co-culture. Additionally, the two populations sorted from the healthy donors’ PBMCs, aforementioned, were cultured separately with MCF-7 spheroids (1:1). This co-culture was also maintained for 4 days, after which the spheroids were collected, stained, and the viability of MCF-7 cells in each condition was assessed as described above.

### 2.5. ELISA

The supernatants from the co-cultures were collected and frozen at −20 °C after centrifugation at 2000 rpm for 10 min. They were used to quantify IFN-γ by ELISA (Biolegend, San Diego, CA, USA) according to the manufacturer’s instructions. Cytokine concentration was calculated using the specific standard curves.

### 2.6. Statistical Analysis

Statistical analysis was performed in GraphPad Prism v6 and SPSS v25 (IBM, Armonk, NY, USA). Comparison between samples was performed by nonparametric Mann–Whitney test. To assess the biomarker performance, ROC curves were performed to assign a threshold to divide NACT-responders from non-responders. This cut-off point corresponded to the maximum of sensitivity and specificity. Univariate and multivariate logistic regressions were conducted taking into account both cohorts in the same analysis. Progression-free survival was analyzed by a Kaplan–Meier curve with a log-rank test and hazard ratio analysis. The probability model was elaborated in R [22]. Statistical significance was considered for *p* < 0.05.

## 3. Results

### 3.1. Clinical Validation of HLA-DR Level in Cytotoxic T Cells (CTLs) as a Predictive Biomarker of Response to NACT

The characteristics of the patients selected to performed NACT (1st cohort [9] and 2nd cohort, flowchart in Figure 1) are described in Table 1.

**Figure 2:**
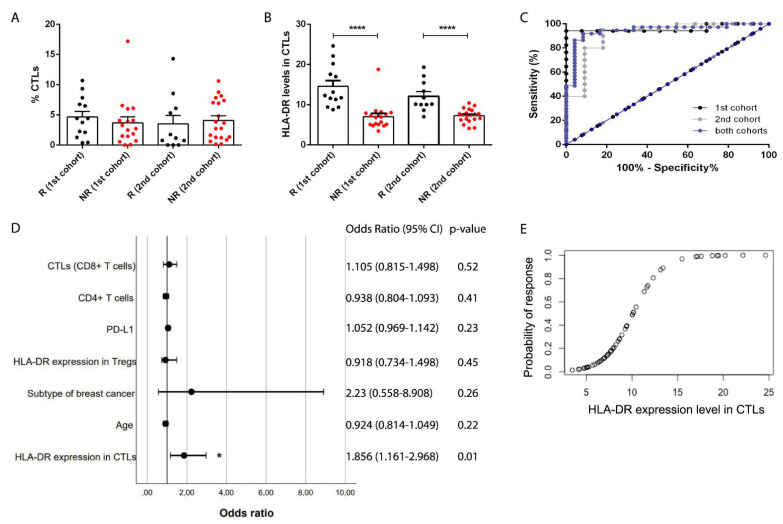
HLA-DR level in cytotoxic T cells is a clinically validated predictive biomarker of breast cancer patients’ response to NACT. (**A**) Percentage of infiltrating cytotoxic T cells (CTLs) in NACT-responders (black dots, 1st cohort *n* = 13 and 2nd cohort *n* = 11) and non-responders (red dots, 1st cohort *n* = 17 and 2nd cohort *n* = 20) breast cancer patients. (**B**) HLA-DR expression level in CTLs in the same patients as in (**A**). (**C**) ROC curve analysis of HLA-DR level in CTLs for the 1st cohort (black lines), 2nd cohort (grey lines), and merged cohorts (blue lines). (**D**) Forest plot of the multivariate analysis performed with both cohorts, including the odds ratio (with 95% confidence interval) and the *p*-values. (**E**) Predictive probability model of response to NACT according to the HLA-DR level in CTLs (merged cohorts). * *p* < 0.05, ****p < 0.0001.

We have previously observed, in the pilot study with 30 breast cancer (BC) patients selected for NACT, that HLA-DR level in CTLs was a putative predictive biomarker for the response to this treatment [9]. Following the REMARK criteria [20], we have enrolled 31 patients in a 2nd independent cohort to validate this biomarker (Figure 2). Response to NACT was defined as before [9]. As in the first cohort, in this new cohort, although the percentage of total CTLs in the biopsies was identical between NACT-responders and non-responders (Figure 2A), patients with response to NACT had higher expression of HLA-DR in intratumor CTLs when compared to NACT non-responders (*p* < 0.0001, Figure 2B and Figure S2). Then, following the REMARK criteria, we performed a series of statistical analyses, namely ROC curve and univariate and multivariate regressions.

ROC curve was executed to determine the cut-off point of HLA-DR level in CTLs to segregate NACT-responders from non-responders (Figure 2C). In the ROC curve of the first cohort [9], this value was 8.94, with an area under the curve (AUC) of 0.96, 94.12% sensitivity, and 100% specificity (Figure 2C). Remarkably, in the ROC curve of the 2nd cohort, we obtained similar parameters, namely, a threshold value of 8.63, an AUC of 0.91, 80% sensitivity, and 90.91% specificity (Figure 2C), hence, validating our biomarker as a predictor of response to NACT.

Then, in order to pursue statistical analyses, we pooled the data from both cohorts. The resultant ROC curve had a cut-off value of 9.32, an AUC of 0.95, 91.89% sensitivity, and 91.67% specificity (Figure 2C), which were very strong parameters, thus, corroborating the robustness of HLA-DR level in CTLs to discriminate NACT-responders from non-responders.

By univariate analysis, we observed that the biomarker HLA-DR level in CTLs was the only significant variable in predicting response to NACT (*p* < 0.0001, OR = 1.965 (95% CI 1.35–2.86), Table S1) in a multitude of clinical data and other immunological features assessed. By multivariate analysis (represented by the forest plot in Figure 2D), we observed that this biomarker can predict BC patients’ response to NACT (*p* = 0.01, OR = 1.856 (95% CI 1.161–2.968)), even when the patients’ age, the BC subtype, and other immunological parameters of the tumor, such as HLA-DR expression level in Tregs, PD-L1 expression in tumor cells, and percentage of infiltrating CD4+ and CD8+ T cells, are taken into account.

Since we have previously demonstrated that there was a correlation between the HLA-DR level in systemic and intratumor CTLs [9], here, we used another cohort of BC patients (41 non-matched blood samples) and observed that NACT-responders present higher levels of HLA-DR in circulating CTLs than non-responders (*p* = 0.03, Figure S3), supporting the idea that this biomarker is reflected in circulation. Nonetheless, the ROC curve parameters, in this case, were not so strong as for the intratumor HLA-DR levels in CTLs (cut-off value of 15.72, AUC of 0.7, 85% of sensitivity, and 52.38% of specificity).

Therefore, anticipating a potential clinical implementation of this biomarker to determine which patients will benefit from NACT and which should be promptly directed to surgery (if possible) or to alternative treatments, we have developed a predictive probability model, based on the analysis of this biomarker in BC patients’ biopsies (Figure 2E), to guide wiser therapeutic decisions. Indeed, following the equation:

(1) $$P\left(R\right)=\frac{1}{1+e^{-\left(-6.49+0.64\times HLA-DR\right)}}\times 100$$

where P(R) is the probability of response, and HLA-DR is the level of this marker in CTLs, it is possible to assess, in advance, the likelihood of a BC patient to respond to NACT, considering the biomarker here described.

### 3.2. HLA-DR Level in CTLs Is also a Prognostic Factor for Breast Cancer

**Figure 3:**
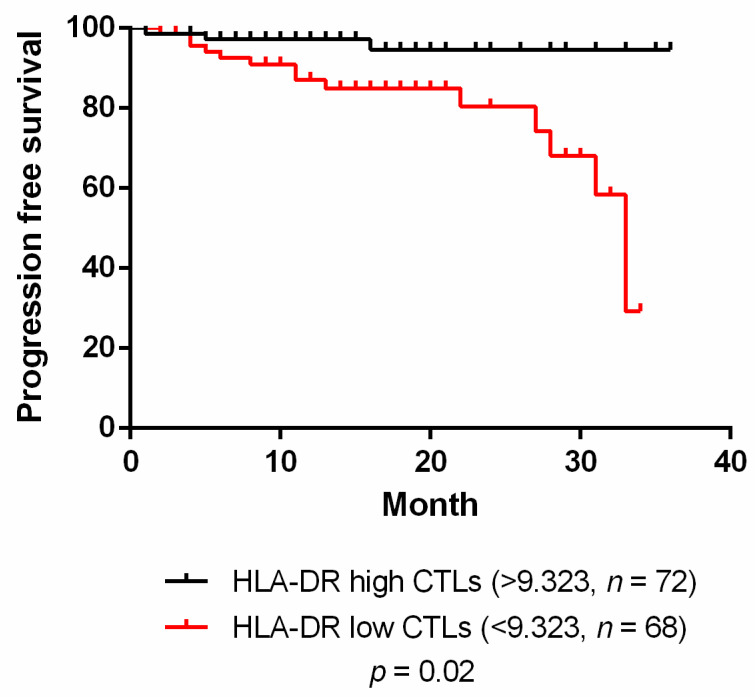
HLA-DR level in cytotoxic T cells has prognostic value. Progression-free survival of breast cancer patients divided in HLA-DR high in cytotoxic T cells (CTLs) (black line, *n* = 72) and HLA-DR low in CTLs (red line, *n* = 68); this segregation was performed according to the threshold value given by the ROC curve with data from both cohorts.

Since the expression level of HLA-DR in CTLs is related to the activation status of these cells, we hypothesized that besides being a predictive biomarker of NACT response, it could also be useful as a prognostic factor, related to patients’ clinical outcome. As such, we have enrolled both non-NACT and NACT patients and performed a follow-up of 34 months. Dividing patients with HLA-DR^low^ CTLs and with HLA-DR^high^ CTLs (assessed in the biopsies or surgical specimens) according to the threshold value calculated in the ROC curve (considering both cohorts, Figure 2C), we observed a significant relationship between the level of HLA-DR in CTLs and the progression-free survival (PFS) curve (*p* = 0.02, Figure 3). Namely, patients with HLA-DR^low^ CTLs have a lower PFS (hazard ratio (HR) = 4.98 (95% CI 1.54–10.31)) when compared to patients with HLA-DR^high^ CTLs (HR = 0.2 (95% CI 0.1–0.65), Figure 3). Thus, we propose that patients with HLA-DR^low^ CTLs progress or relapse sooner than patients with HLA-DR^high^ CTLs, regardless of the treatment.

A similar analysis was performed for overall survival (OS), but no differences were found in this case (data not shown), probably because for OS, a longer follow-up should be performed.

### 3.3. HLA-DR+ CTLs Express High Levels of Activation and Proliferation Markers, Release Effector Molecules, and Are Cytotoxic against Tumor Cells

**Figure 4:**
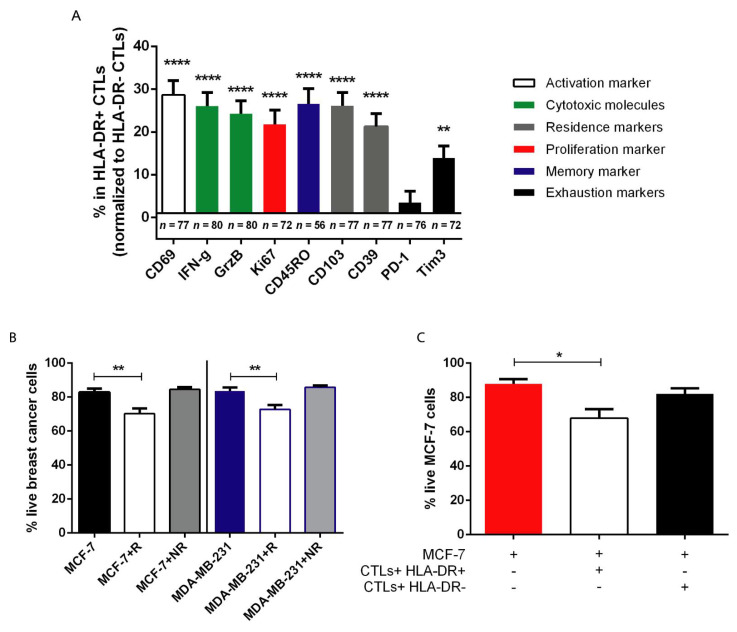
HLA-DR+ CTLs have enhanced anti-tumor properties in comparison to HLA-DRnegative CTLs. (**A**) Percentage of the activation marker CD69, the cytotoxic molecules IFN-γ and Granzyme B (GrzB), the proliferation marker Ki67, the memory marker CD45RO, the tissue-resident markers CD103 and CD39, and the exhaustion markers PD-1 and Tim3 within the tumor infiltrating population of HLA-DR+ CTLs, normalized to the percentage of these markers within the tumor infiltrating population of HLA-DRnegative CTLs (number of biopsies used for this characterization is described in the graph). (**B**) Percentage of live MCF-7 cells in monoculture (MCF-7, black bar, *n* = 10), in co-culture with NACT-responders’ PBMCs (MCF-7+R, white bar, *n* = 10), and with NACT non-responders’ PBMCs (MCF-7+NR, grey bar, *n* = 11); percentage of live MDA-MB-231 cells in monoculture (MDA-MB-231, blue bar, *n* = 11), in co-culture with NACT-responders’ PBMCs (MDA-MB-231+R, white bar, *n* = 10), and with NACT non-responders’ PBMCs (MDA-MB-231+NR, grey bar, *n* = 12). (**C**) Viability of MCF-7 cells either in monoculture (red bar, *n* = 5) or in co-culture with sorted HLA-DR+ CTLs (white bar, *n* = 8) and HLA-DRnegative CTLs (black bar, *n* = 8). Data are represented as mean ± SEM, *p < 0.05, **p < 0.01, ****p < 0.0001.

To shed some light on the reason why higher levels of HLA-DR in the tumor-infiltrating CTLs are required for the achievement of a good response to NACT, and eventually a better prognosis, we tried to better characterize the profile of HLA-DR+ CTLs in comparison with HLA-DRnegative CTLs (Figure 4A). By flow cytometry, we observed that, when compared to HLA-DRnegative CTLs, HLA-DR+ CTLs were more activated (CD69, *p* < 0.0001), and proliferative cells (Ki67, *p* < 0.0001) that produced more cytotoxicity-related molecules (IFN-γ and Granzyme B, *p* < 0.0001) showed tissue-residency (CD103 and CD39, *p* < 0.0001) and memory (CD45RO, *p* < 0.0001) features with similar levels of PD-1 and increased expression of Tim3 (*p* < 0.01).

Then, and since we demonstrated that in blood from NACT-responders, CTLs also exhibit higher levels of HLA-DR than CTLs from NACT non-responders ([9], Figure S3) we used patient-derived peripheral blood mononuclear cells (PBMCs) isolated from the blood to perform ex vivo assays.

With the 3D in vitro platform we have developed [21], we added to two different BC cell lines (MCF-7 and MDA-MB-231), PBMCs from NACT-responders and non-responders and assessed the viability of the tumor cells when in contact with patient-derived PBMCs (Figure 4B). Interestingly, we noticed that the addition of NACT-responders’ PBMCs (that are enriched in HLA-DR^high^ CTLs (Figure S3)) to the BC 3D-structure reduced their viability after 4 days in co-culture (*p* = 0.04 and *p* = 0.06, Figure 4B); whereas the addition of non-responders’ PBMCs (that lack HLA-DR^high^ CTLs) showed no effect on the viability of the BC cells (Figure 4B), recapitulating the clinical observations (Figure 2 and Figure S3).

Taking into consideration that HLA-DR+ CTLs are more activated, proliferative, and produce more cytotoxicity-related molecules (Figure 4A), we hypothesized that HLA-DR+ CTLs indeed exhibit anti-tumor properties and are cytotoxic against tumor cells, unlike HLA-DRnegative CTLs. To confirm this, we used sorted HLA-DR+ and HLA-DRnegative CTLs, isolated from the same healthy individuals after stimulation of their PBMCs, and cultured them separately with 3D structures of MCF-7 BC cells (Figure 4C). After 4 days in co-culture, we observed that the BC viability was reduced only in the presence of HLA-DR+ CTLs (*p* = 0.02, Figure 4C), emphasizing that these cells, and not HLA-DRnegative CTLs, are cytotoxic against tumor cells.

### 3.4. Stimulation of HLA-DR Level in CTLs Could Increase their Anti-Tumor Properties

**Figure 5:**
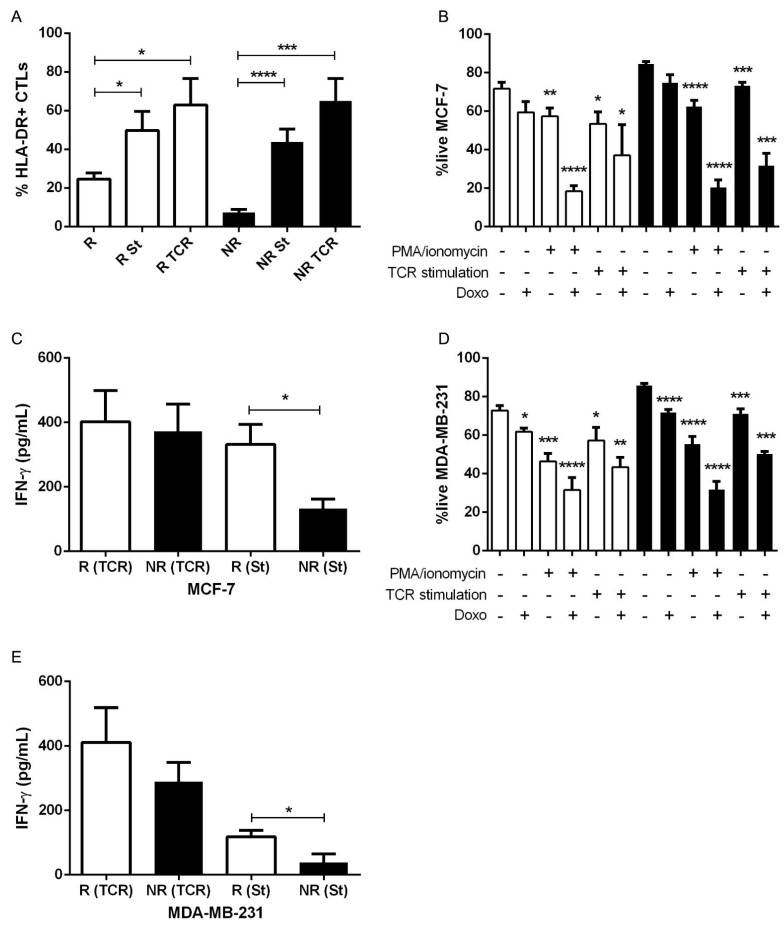
NACT non-responders’ PBMCs previously stimulated with PMA/ionomycin or by TCR engagement are capable of reducing the breast cancer cells’ viability. (**A**) Percentage of HLA-DR+ CTLs in PBMCs of NACT-responders (R, white bar, *n* = 12), NACT-responders with a canonical stimulation of PMA/ionomycin (R St, white bar, *n* = 10), NACT-responders with TCR stimulation (R TCR, white bar, *n* = 4), NACT non-responders (NR, black bar, *n* = 11), NACT non-responders stimulated with PMA/ionomycin (NR St, black bar, *n* = 12), and NACT non-responders with TCR stimulation (NR TCR, black bar, *n* = 6). (**B**) Viability of MCF-7 cells in co-culture with NACT-responders’ PBMCs (R, white bars, *n* = 4–11) or with NACT non-responders’ PBMCs (NR, black bars, *n* = 6–11) in the presence/absence of doxorubicin (Doxo), with or without previous canonical stimulation (PMA/ionomycin) and with or without previous TCR stimulation. (**C**) IFN-γ production of stimulated NACT-responders’ PBMCs with PMA/ionomycin (R (St), white bar, *n* = 11) or with TCR stimulation (R (TCR), white bar, *n* = 4); and stimulated NACT non-responders’ PBMCs with PMA/ionomycin (NR (St), black bar, *n* = 12) or with TCR stimulation (NR (TCR), black bar, *n* = 6) after 4 days of co-culture with MCF-7 cells. (**D**) Viability of MDA-MB-231 cells in co-culture with NACT-responders’ PBMCs (R, white bars, *n* = 4–10) or with NACT non-responders’ PBMCs (NR, black bars, *n* = 6–12) in the presence/absence of doxorubicin (Doxo), with or without previous canonical stimulation (PMA/ionomycin) and with or without previous TCR stimulation. (**E**) IFN-γ production of stimulated NACT-responders’ PBMCs with PMA/ionomycin (R (St), white bar, *n* = 6) or with TCR stimulation (R (TCR), white bar, *n* = 4); and stimulated NACT non-responders’ PBMCs with PMA/ionomycin (NR (St), black bar, *n* = 9) or with TCR stimulation (NR (TCR), black bar, *n* = 6) after 4 days of co-culture with MDA-MB-231 cells. Data are represented as mean ± SEM, *p < 0.05, **p < 0.01, ***p < 0.001, ****p < 0.0001.

Considering that CTLs expressing a high level of HLA-DR in the tumor microenvironment (and consequently in the blood) are a prerequisite for NACT success, probably due to their capacity to decrease the viability of tumor cells, we intended to further explore the therapeutic potential of these cells, aiming to improve conventional BC treatment. Therefore, we stimulated patients’ PBMCs to increase HLA-DR in CTLs before adding these cells to the cancer spheroid. To achieve this stimulation, we used two different strategies—the canonical PMA/ionomycin stimulation and the TCR engagement. With both methods, the percentage of HLA-DR+ CTLs increased in both NACT-responders (*p* = 0.04 and *p* = 0.01 for PMA/ionomycin and TCR stimulation, respectively) and non-responders’ PBMCs (*p* < 0.0001 and *p* = 0.0002 for PMA/ionomycin and TCR stimulation, respectively, Figure 5A and Figure S4). The addition of stimulated PBMCs from non-responders, to either MCF-7 (*p* < 0.0001 and *p* = 0.0003 for PMA/ionomycin and TCR stimulation, respectively, Figure 5B) or MDA-MB-231 spheroids (*p* < 0.0001 and *p* = 0.0001 for PMA/ionomycin and TCR stimulation, respectively, Figure 5D), resulted in a decreased viability of BC cells, suggesting that ex vivo modulation of PBMCs from non-responders, with the consequent increase of the numbers of CTLs expressing high levels of HLA-DR, could indeed ameliorate the anti-tumor ability of these cells.

Additionally, to understand if the combination of chemotherapy and this immune-modulation is advantageous in the reduction of the BC viability, we used the NACT-agent doxorubicin. Interestingly, doxorubicin alone did not show a significant impact on tumor cells viability, especially for the MCF-7 cell line, which could be explained by the higher chemoresistance of this cell line (Figure 5B,D). Nevertheless, the employment of doxorubicin in combination with non-responders’ PBMCs previously stimulated led to a synergistic reduction on the percentage of live tumor cells (*p* < 0.0001 for PMA/ionomycin stimulation in both cell lines and *p* = 0.0002 for TCR stimulation in both cell lines, Figure 5B,D).

Another readout of the anti-tumor ability of the stimulated cells was the concentration of IFN-γ released to the culture supernatant. Indeed, stimulated PBMCs secrete IFN-γ (Figure 5C,E), which in the case of TCR engagement, was even similar in non-responders’ PBMCs and in NACT-responders’ PBMCs (Figure 5C,E). It is noteworthy that while non-stimulated PBMCs of NACT-responders’ produce basal levels of IFN-γ, PBMCs from non-responder patients did not secrete this cytokine into the culture medium (data not shown). These results corroborate the idea that it is possible to alter the phenotype of PBMCs from NACT non-responder patients, therefore, increasing the HLA-DR level in CTLs but also empowering them with anti-tumor capacity. Hence, stimulation of HLA-DR in CTLs could have therapeutic potential, as CTLs expressing high level of HLA-DR might contribute to decrease the resistance of BC tumors to standard treatment.

## 4. Discussion

Biomarkers that can effectively predict breast cancer (BC) patients’ response to neoadjuvant chemotherapy (NACT) have been widely searched for due to the unmet clinical need to determine which patients would not benefit from this treatment and should be promptly directed to more targeted approaches, avoiding the chemotherapy-related toxicity and the misuse of resources. Tumor-infiltrating lymphocytes (TILs) have been studied as predictive biomarkers of the response to NACT [23,24,25,26]. However, TILs assessment is still not used in the clinical routine, despite an international effort to standardize the evaluation of this biomarker [27]. The main disadvantage of the employment of TILs is the fact that they are presented as a single population, though they comprise immune cells with opposite roles (pro- and anti-tumor T cells). Indeed, TILs represent a heterogeneous population, including the immunosuppressive regulatory T cells. Additionally, tumors can escape the immune system through, for instance, the release of anti-inflammatory cytokines (such as IL-10 or TGF-β) or the activation of immune checkpoints, namely, the PD-1/PD-L1 axis, which will prevent the activation of CTLs and, consequently, lower their cytotoxic function. Only when these escape mechanisms are not yet engaged can CTLs become activated by recognizing tumor antigens and produce effector molecules, such as Perforin and Granzyme B, that lead to tumor cell elimination. Moreover, TILs have been suggested mainly for TNBC patients (excluding the majority of BC cases); its analysis is based on immunohistochemistry (IHC) of a single tumor plane, excluding the whole 3D conformation, and similarly, TIL scoring is more subjective.

To overcome the limitations of TILs, we previously proposed [9] and validated in the current study a new biomarker—HLA-DR level in CTLs, which accurately discriminates NACT-responders from non-responders and better reflects the overall immune status of the tumor microenvironment. Actually, HLA-DR+ CTLs found in the biopsies represent a population of activated CTLs, as HLA-DR *per se* is an activation marker of T lymphocytes [13] but also expresses more of other activation markers (e.g., CD69) when compared with HLA-DRnegative CTLs. This subset also has higher proliferative capacity, more tissue-residency and memory markers (which have been highlighted as an interesting anti-tumor phenotype [28]), a similar level of exhaustion, and notably, increased cytotoxic properties against tumor cells (as observed in the 3D co-cultures) when compared to other CTLs present in the tumor microenvironment. Thus, we verified, in two independent cohorts, that HLA-DR level in CTLs is, statistically, by the ROC curve parameters and the univariate and multivariate analysis of the merged data, a powerful predictive factor to segregate BC patients that will or will not respond to NACT. Additionally, this biomarker is independent of the BC subtype and other clinical parameters, such as the patients’ age, reflecting the capacity to be used in all BC patients that would be, based on the current criteria, selected to receive NACT.

Here, we proposed the assessment of HLA-DR expression level in CTLs by flow cytometry and validated its determination in the following conditions: biopsies should be collected in Transfix (to preserve cellular antigens), processed, and stained using a combination of two monoclonal mouse anti-human antibodies, specifically anti-CD8-PE (clone HIT8a) and anti-HLA-DR-APC (clone L243) from Biolegend in a 1:50 concentration. The flow cytometry analysis should be performed by gating HLA-DR within the CD8+ population (CTLs), and the value HLA-DR level in CTLs was obtained by determining the ratio between the median fluorescence intensity (MFI) of HLA-DR+ and the MFI of HLA-DRnegative population, as we previously reported [9]. The employment of flow cytometry could be an advantage because we cannot distinguish intratumor CTLs from stromal CTLs, as it is possible with IHC; hence, this technique is more representative of the whole biopsy than IHC, where only a slice of the 3D structure of the biopsy is used. Actually, several reports claim that stromal TILs are more important than the tumor-infiltrating ones to predict response to NACT (reviewed in [29]), while others report the opposite [30]. With flow cytometry, we can quantify both simultaneously. Nevertheless, flow cytometry is not as widely available in pathology departments of hospitals and clinics as IHC is, which might represent a limitation. Another advantage of the proposed biomarker is its systemic reflection, as NACT-responders also have higher expression of HLA-DR in blood CTLs then non-responders. Nonetheless, the parameters of the ROC curve using blood samples were not as noticeable as for the biopsies; hence, the prediction of NACT response should be assessed in the tissue biopsies. However, blood analysis could be useful as a complement and/or can be performed, for instance, to monitor NACT efficiency throughout the treatment.

Envisioning the application of this biomarker in a clinical setting, we have developed a probability model that can be used by clinicians in the therapeutic decision process. Based on this model, we suggest segregating patients into three groups—high probability of response to NACT (>60% that corresponds to an HLA-DR level higher than 10.8), intermediate probability of response (20–59% that corresponds to an HLA-DR between 8 and 10.8), and low probability of response (<20% that corresponds to an HLA-DR level lower than 8). This stratification should help to guarantee that patients with high probability of response to NACT will be submitted to this treatment, while patients with low probability of response to NACT will be promptly directed to surgery/alternative therapies whenever possible. Of course, for the cases of patients with intermediate probability of response, the therapeutic decision would be more challenging, and clinicians should analyze it case-by-case and contemplate different aspects, such as if another therapeutic option is available or if NACT is still the best option. It is noteworthy that the equation presented was extrapolated from the results of these studies, using both cohorts merged, so it is only valid for HLA-DR level in CTLs ranging from 4.13 to 24.63 when determined as we did.

In addition to being a predictive factor, HLA-DR level in CTLs is also associated with the progression-free survival curve considering 34 months of follow-up of all the patients whose biopsies/surgical specimens we analyzed, independently of the treatment performed. This observation suggests that besides the predictive value of this biomarker for the response to a specific treatment, it has also a promising prognostic value that could be useful to determine, in advance, BC patients’ outcome.

Recently, we have developed a 3D platform composed of BC cell lines and PBMCs [21], anticipating the use of this system as a patient-customized drug screening assay. Here, we took advantage of this 3D system to demonstrate in vitro that only PBMCs from NACT-responders have the ability to reduce the viability of tumor cells. Given the fact that HLA-DR expression is boosted in circulating CTLs from NACT-responder patients, we further showed that it is this subset, and not other CTLs, that in fact are responsible for the anti-tumor properties of NACT-responders’ PBMCs. In line with the idea that an immune status favoring cytotoxicity against tumors will determine the success of chemotherapy by allowing the control of chemotherapy-resistant cancer cells, these results contribute to explain the requirement of CTLs expressing high levels of HLA-DR for NACT efficacy and a good outcome, corroborating clinical observations.

The identification of patients who most likely will not respond to NACT is critical to promptly direct them for alternative and/or individualized treatments, although, so far, these treatments are still scarce. Given the observation that CTLs with enhanced expression of HLA-DR have anti-tumor properties and are important for NACT success, to tackle this issue, we used our 3D co-culture assay also to investigate the therapeutic potential of these cells. Excitingly, the results showed that the stimulation of NACT non-responders’ immune cells raises HLA-DR level at the surface of their CTLs and, therefore, increases the ability of these cells to reduce the viability of cancer cells in culture. This effect was even more pronounced when stimulated immune cells were added to the cancer cells in the presence of doxorubicin, suggesting that the upsurge of HLA-DR level in CTLs, especially of NACT non-responders, could be a promising therapeutic strategy to combine with chemotherapeutic agents to ameliorate BC treatments.

## 5. Conclusions

NACT is a widely accepted therapeutic option for patients with locally advanced BC. However, in patients with chemotherapy-resistant tumors, it is not efficient and, in some cases, leads to even more severe forms of the disease. Here, we validated, in an independent cohort, HLA-DR level in CTLs as a simple and robust biomarker to determine the likelihood of tumor remission after NACT and developed a predictive probability model foreseeing its implementation in the clinical routine. This biomarker also has the potential to be used in clinical trial design to evaluate highly expensive and technically demanding immunotherapies, particularly in those BC patients who might truly need/take advantage of these novel treatments. In the future, it may be also adapted as a predictive/prognostic factor for other types of cancer.

The lack of alternative therapeutic options for BC patients with modest response to conventional treatments is another obstacle that needs to be circumvented. Actually, the discovery of new predictive biomarkers should go hand-in-hand with the development of new treatments, to be able to offer to patients that will not respond to conventional therapies. Thus, here, we also showed that it is possible to modulate the expression of HLA-DR in CTLs and, consequently, improve the ability of these cells to kill tumor cells in culture, opening new therapeutic possibilities based on these cells. Nonetheless, more clinical grade immune-modulators to increment HLA-DR in patients’ CTLs should be further explored to translate this approach for BC patients and improve the management of this disease.

## Figures and Tables

**Table 1:** Characteristics of patients enrolled in this study (median values of age, body mass index, and percentage of post-menopause patients). Clinical data, such as subtype of breast cancer, grade, median of Ki67, and of tumor dimension and node status, are also described.

Characteristics	1st Cohort		2nd Cohort	
	Responders (*n* = 13)	Non-Responders (*n* = 17)	Responders (*n* = 11)	Non-Responders (*n* = 20)
Age (median + range)	57 (39–82)	64 (45–79)	47.5 (29–67)	58 (39–71)
Body mass index (median + range)	25.31 (22.04–32.03)	25.39 (22.07–33.2)	32.05 (20.51–46.64)	28.01 (19.36–39.73)
Post-menopause	76.92%	58.82%	45.45%	77.78%
ER+ (PR –/+)	30.77%	41.18%	11.12%	47.37%
HER2+ including triple positive breast cancer	38.46%	17.64%	44.44%	42.10%
TNBC	30.77%	41.18%	44.44%	10.53%
Grade	G1—15.38%	G1—0%	G1—25%	G1—17.65%
	G2—30.77%	G2—47.06%	G2—37.5%	G2—47.06%
	G3—53.85%	G3—52.94%	G3—37.5%	G3—35.29%
Ki67 (median + range)	47.40% (15–97)	19.15% (2–80)	40% (10–75)	40% (5–90)
Tumor dimension in mm (median + range)	38 (19–63)	25 (10–70)	29.5 (7–65)	25 (9–80)
Axillary lymph node invasion status	Negative—38.46%	Negative—35.29%	Negative—44.44%	Negative—23.53%
	Positive—61.54%	Positive—64.71%	Positive—55.56%	Positive—76.47%

## Data Availability

Not applicable.

## Supplementary Materials

The following are available online at https://www.mdpi.com/article/10.3390/cancers13153841/s1: Figure S1: Gating strategy. Figure S2: Flow cytometry dot plot of HLA-DR gated inside CD3+/CD8+ in biopsies of NACT non-responders and NACT-responders. Figure S3: HLA-DR expression level in systemic cytotoxic T cells segregate breast cancer patients according to their response to neoadjuvant chemotherapy. HLA-DR expression level in circulating cytotoxic T lymphocytes (CTLs) of NACT-responders (R, black dots, *n* = 21) and NACT non-responders (NR, red dots, *n* = 20). **p* < 0.05. Figure S4: Flow cytometry dot plot of HLA-DR gated inside CD3 + /CD8+ in PBMCs isolated from breast cancer patients with or without previous PMA/ionomycin stimulation. Table S1: Univariate analysis for the biomarker HLA-DR level in CTLs. HLA-DR expression level in CTLs and other immunological markers and clinical parameters were analyzed by univariate analysis. *p*-value, odds ratio and the confidence interval of the odds ratio are represented.
