# Supplementary material for: Expression of HLA-DR in Cytotoxic T Lymphocytes: A Validated Predictive Biomarker and a Potential Therapeutic Strategy in Breast Cancer
Source: Cancers (Basel). 2021 Jul 30;13(15):3841. doi: 10.3390/cancers13153841 (PMC8345089; doi:10.3390/cancers13153841)
Supplement: Supplementary file 1 [file cancers-13-03841-s001.zip › cancers-1267370-supplementary.pdf]

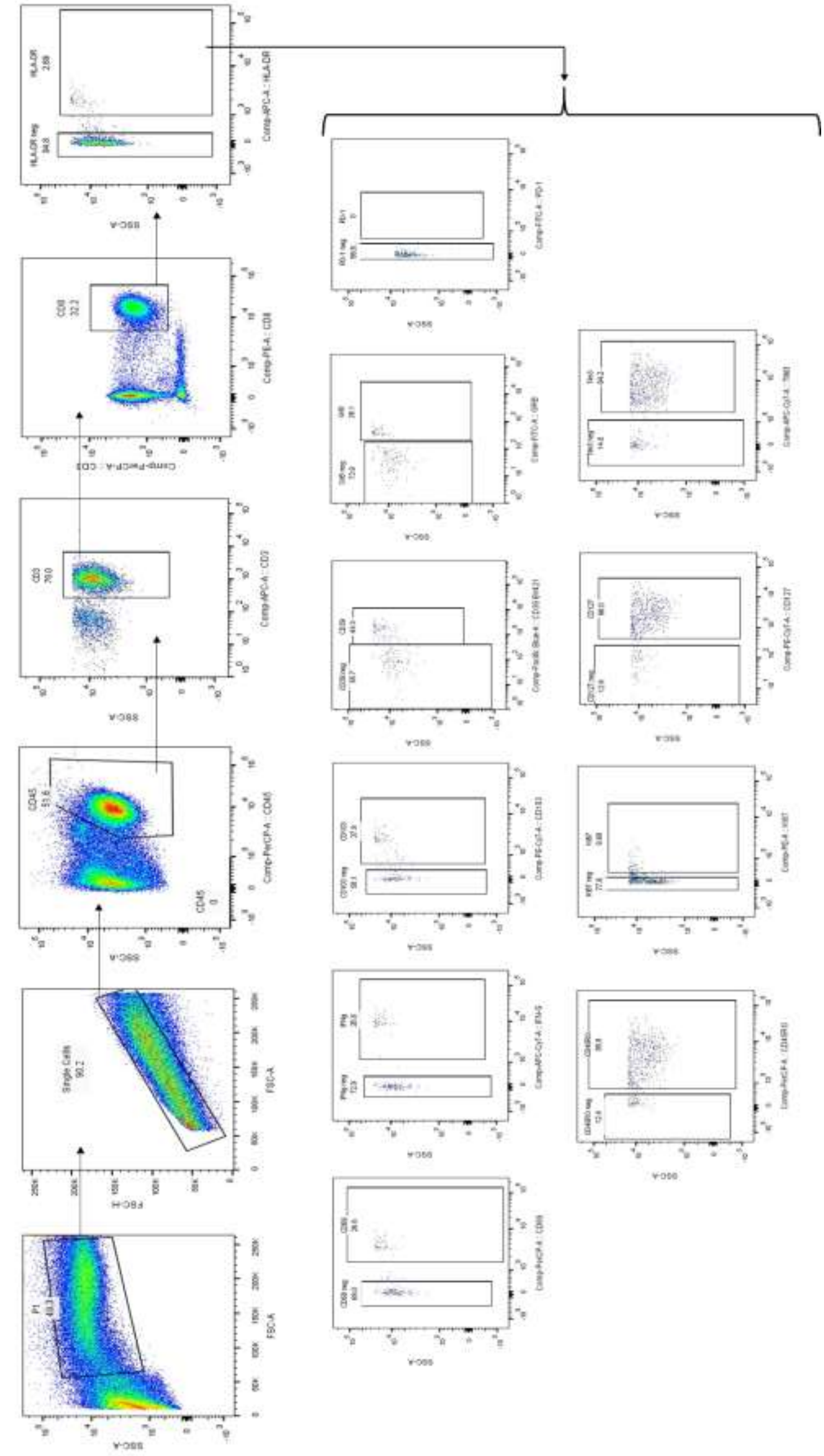

Figure S1. Gating strategy.

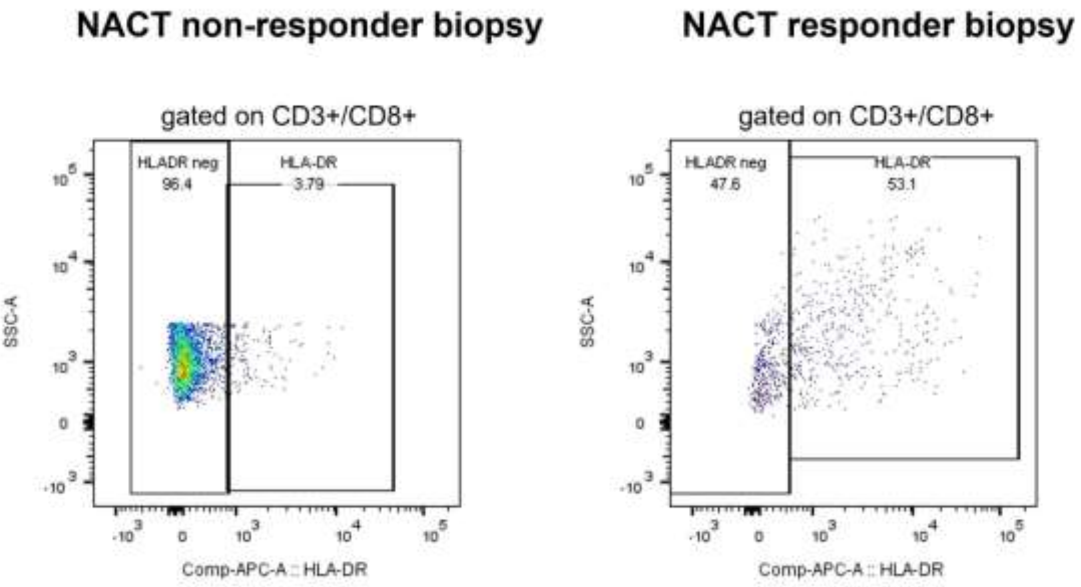

Figure S2. Flow cytometry dot plot of HLA-DR gated inside CD3+/CD8+ in biopsies of NACT non-responders and NACT-responders.

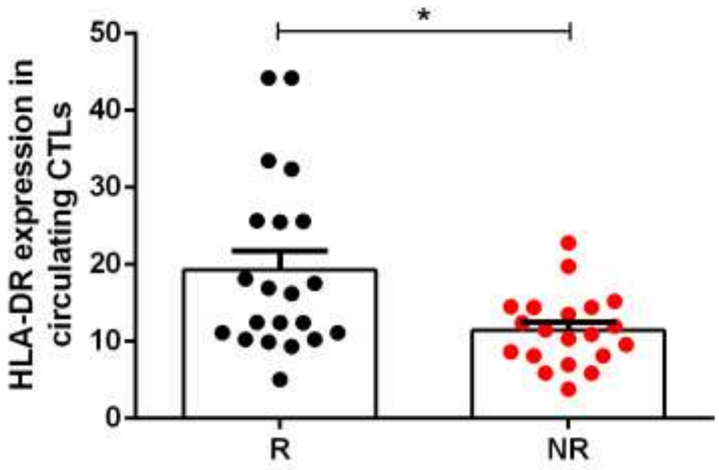

Figure S3. HLA-DR expression level in systemic cytotoxic T cells segregate breast cancer patients according to their response to neoadjuvant chemotherapy. HLA-DR expression level in circulating cytotoxic T lymphocytes (CTLs) of NACT-responders (R, black dots, $n = 21$) and NACT non-responders (NR, red dots, $n = 20$). *$p < 0.05$.

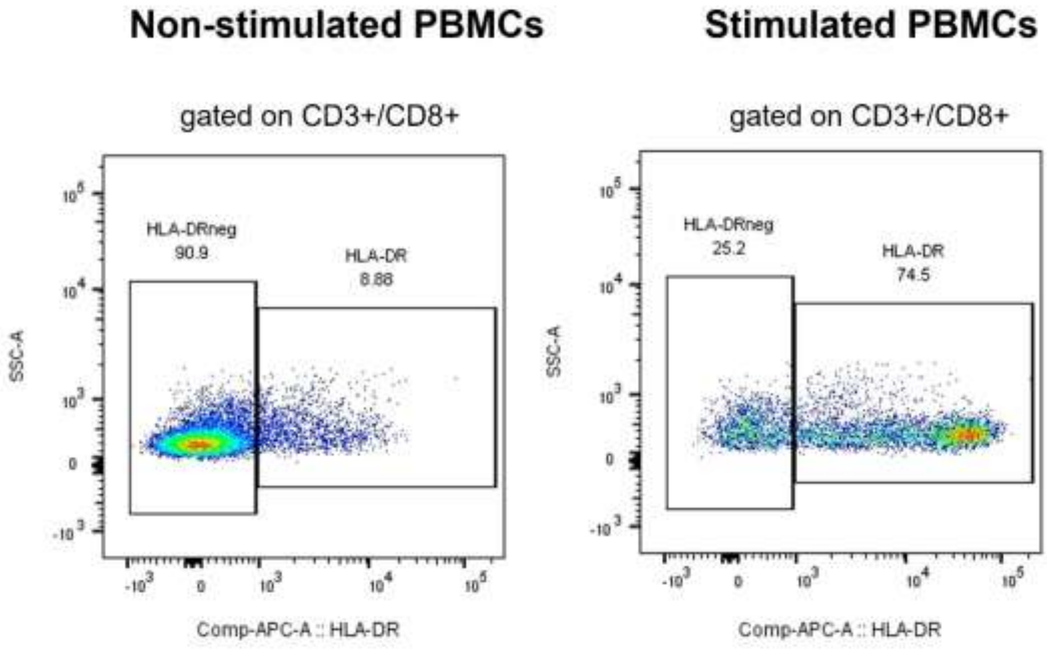

Figure S4. Flow cytometry dot plot of HLA-DR gated inside CD3+/CD8+ in PBMCs isolated from breast cancer patients with or without previous PMA/ionomycin stimulation.

Table S1. Univariate analysis for the biomarker HLA-DR level in CTLs. HLA-DR expression level in CTLs and other immunological markers and clinical parameters were analyzed by univariate analysis. *p*-value, odds ratio and the confidence interval of the odds ratio are represented.

Parameter	*p*-value	Odds Ratio	95% Confidence Interval
HLA-DR expression level in CTLs	<0.0001	1.965	1.35–2.86
Immune cells (CD45)	0.985	1.000	0.965–1.036
T cells (CD3)	0.520	0.985	0.94–1.032
B cells (CD19)	0.919	0.995	0.904–1.095
NK cells (CD161)	0.122	0.852	0.696–1.044
T helper cells (CD4)	0.282	0.972	0.922–1.024
CTLs (CD8)	0.934	1.006	0.875–1.156
Tregs ($CD25^{high}$/$CD127^{low}$)	0.503	1.157	0.755–1.772
M1 macrophages (CD11b+/CD163-/CD206-)	0.719	0.628	0.05–7.944
M2 macrophages (CD11b+/CD163+/CD206+)	0.743	0.939	0.644–1.369
Neutrophils (CD15)	0.298	0.897	0.731–1.101
HLA-DR expression level in Tregs	0.724	0.981	0.885–1.089
PD-L1	0.165	1.015	0.994–1.037
IL-10	0.107	1.051	1–1.104
Age	0.254	0.975	0.933–1.018
Body Mass Index	0.69	0.977	0.869–1.097
Tumor dimension	0.167	1.024	0.99–1.06
Node invasion status	0.338	0.566	0.177–1.813
Ki67 (%)	0.271	1.012	0.991–1.033
Grade	0.474	0.563	0.116–2.718
